# Epidemic Urticaria

**Published:** 1882-10-20

**Authors:** 


### *EPIDEMIC URTICARIA.*

Urticaria, or nettle-rash, is so prevalent in Upper Georgia that some have considered it epidemic. This would not be more strange than the epidemic of Whitlow which prevailed all over the United States in or about the year 1850.

A good treatment for urticaria in its acute form is a purgative dose of cream of tartar and sulphur, followed by low diet for a day or two. If periodic in character quinine should be given.

